# The Role of ATG8 in Promoting Lipid Accumulation in the Oleaginous Fungus *Mucor circinelloides* During Nitrogen Limitation

**DOI:** 10.3390/jof12060410

**Published:** 2026-06-04

**Authors:** Hequn Li, Hongjuan Yuan, Bushra Iqbal, Tianyu Wang, Zhen Wang, Huaiyuan Zhang

**Affiliations:** 1 Colin Ratledge Center for Microbial Lipids, College of Agricultural Engineering and Food Science, Shandong University of Technology, 266 Xincun West Road, Zibo 255000, China; 19811715276@163.com (H.L.); y17663072167@163.com (H.Y.); bushra.iqbal1212@gmail.com (B.I.); 19819139268@163.com (T.W.); 2 School of Public Health, Qilu Medical University, Zibo 255300, China

**Keywords:** *Mucor circinelloides*, lipid accumulation, autophagy, ATG8, oleaginous microorganism

## Abstract

Autophagy is a central cellular process that recycles intracellular components and supplies precursors for biosynthesis. As a key regulator of autophagosome formation, autophagy-related protein 8 (ATG8) plays an essential role in macromolecular degradation and in the availability of lipid precursors. However, whether enhanced autophagic flux promotes lipid accumulation in oleaginous fungi remains unclear. In this study, *atg8-1* and *atg8-2* were homologously overexpressed in the oleaginous fungus *Mucor circinelloides* to evaluate their roles in lipid biosynthesis. The engineered strains McATG8-1T2 and McATG8-2T2 showed significantly increased total fatty acid (TFA) contents (32.9% and 32.5%), representing improvements of 15.0% and 13.7% compared with the control. γ-Linolenic acid levels were also elevated to 16.9% and 16.5%, relative increases of 25.2% and 22.0%, respectively. RT-qPCR analysis revealed coordinated upregulation of genes involved in autophagy, central carbon metabolism, lipid biosynthesis, and the pentose phosphate pathway. Ethanolamine supplementation further enhanced lipid accumulation, increasing TFA contents by 12.2–14.6%. In addition, inhibition of target of rapamycin complex 1 using rapamycin produced a strong synergistic effect with *atg8* overexpression, leading to substantial lipid increases under nitrogen-limited and nitrogen-rich conditions. Collectively, these findings demonstrated that ATG8-mediated autophagy enhanced lipid accumulation and acted as a key determinant of lipid synthesis flux.

## 1. Introduction

Microbial oils have attracted considerable attention as sustainable sources of polyunsaturated fatty acids (PUFAs) due to their short production cycles and relatively low environmental constraints. These oils hold significant potential for applications in the food, nutrition, and fine chemical industries [1,2]. In conventional fermentation processes using oleaginous microorganisms, nitrogen limitation is widely employed to redirect carbon flux toward lipid biosynthesis [3]. However, this strategy presents a fundamental challenge: the temporal mismatch between biomass accumulation and lipid production. While cell growth depends on sufficient nitrogen availability, lipid accumulation is typically triggered under nitrogen-depleted conditions. Thus, lipid synthesis often occurs at the expense of cell proliferation, ultimately constraining overall lipid yield [4,5]. Addressing this trade-off requires a deeper understanding of the cellular signaling and metabolic reprogramming that occur under nitrogen limitation, with the aim of enhancing lipid accumulation without compromising biomass formation.

Autophagy, a highly conserved intracellular degradation system in eukaryotes, plays a central role in maintaining cellular energy homeostasis under nutrient stress [6,7]. Under nitrogen-rich conditions, target of rapamycin complex 1 (TORC1) remains active, promoting anabolic processes while repressing autophagy [8,9]. In contrast, nitrogen starvation leads to TORC1 inhibition and subsequent activation of autophagy [10]. Traditionally viewed as a survival mechanism that recycles cellular components for energy production, autophagy is now being increasingly recognized as a key contributor to metabolic reprogramming. Emerging multi-omics evidence suggests that autophagy facilitates carbon redistribution in oleaginous microorganisms. For example, experimental evidence in *Mortierella alpina* demonstrated that nitrogen limitation triggers autophagy, which is closely associated with the upregulated transcription of key lipogenic genes and enhanced triacylglycerol (TAG) accumulation [11,12,13]. This process effectively channels degradation-derived metabolites into TAG biosynthesis, demonstrating that autophagy is an intrinsic driver of lipid accumulation under stress conditions. Nevertheless, the specific molecular regulators governing this metabolic shift, as well as their potential rate-limiting roles, remain largely unresolved.

Autophagy-related protein 8 (ATG8) is a core component of the autophagic machinery, and is essential for autophagosome membrane expansion and maturation [14,15]. Crucially, its activity depends on covalent conjugation with phosphatidylethanolamine (PE), to form the ATG8-PE complex, which is critical for autophagosome formation and function [16,17]. There is increasing evidence of a close association between ATG8 and lipid metabolism. In *M. alpina*, *atg8* is required for lipid synthesis, and supplementation with ethanolamine, which enhances PE availability and promotes ATG8 lipidation, significantly increases lipid biosynthesis [12]. Similarly, genome-wide analyses in *Rhodosporidium toruloides* have identified autophagy as a key regulatory pathway for lipid accumulation, with ATG8 playing an essential role in maintaining a high lipid content [18,19]. Coordinated upregulation of ATG8-mediated autophagy and lipid droplet-associated genes under nitrogen starvation supports TAG accumulation and ARA synthesis in *Lobosphaera incisa* [20]. Collectively, these results indicate that ATG8 is not merely a structural component of the autophagic machinery, but also functions as a crucial modulator of lipid metabolism.

ATG8 function is closely linked to the TORC1 signaling pathway. In *Saccharomyces cerevisiae*, loss of ATG8 increases cellular sensitivity to the TORC1 inhibitor rapamycin, indicating a functional interplay between ATG8 activity and TORC1 regulation [21]. Lipidation-dependent and -independent roles of ATG8 have been implicated in cellular responses to TORC1 inhibition. Moreover, TORC1 influences autophagy at multiple levels, including phosphorylation of upstream regulators such as ATG13, modulation of ATG mRNA stability, and maintenance of ATG8 protein stability [22,23]. These findings highlight ATG8 as a downstream effector and regulatory node within the TORC1 signaling network. Despite these well-established roles, the mechanisms through which ATG8-mediated autophagy integrates TORC1 signaling to regulate lipid accumulation in oleaginous filamentous fungi remain poorly understood. To address this knowledge gap, for the first time, the role of ATG8 was investigated in the oleaginous filamentous fungus *M. circinelloides*, which, despite having genetic tools, broad biotechnological potential, and the capacity for γ-linolenic acid (GLA) synthesis [24,25], has been less studied regarding the link between autophagy and lipidogenesis than other model organisms. Furthermore, this study explored its interplay with TORC1 signaling and lipid biosynthesis. This work therefore provides new insights into the regulatory networks governing lipid metabolism in oleaginous fungi and may help identify potential strategies for improving microbial lipid production.

## 2. Materials and Methods

### 2.1. Strains and Culture Conditions

The *atg8-1* and *atg8-2* genes were obtained from *M. circinelloides* WJ11 (CCTCC no. M2014424). These genes were overexpressed in the uracil auxotrophic strain M65 (derived from *M. circinelloides* WJ11). This strain was cultivated on yeast extract peptone glycerol (YPG) solid medium (pH 4.5) supplemented with 200 μg/mL uracil to support routine mycelial and colony growth. The recombinant plasmids were propagated in *Escherichia coli* TOP10, which was grown in Luria–Bertani (LB) broth containing 100 μg/mL ampicillin at 37 °C with shaking at 200 rpm for all cloning steps.

### 2.2. Construction of M. circinelloides atg8 Overexpression Strains

Complementary DNA (cDNA) was synthesized from total RNA, extracted from *M. circinelloides* WJ11 cells utilizing Trizol reagent (Sangon Biotech, Shanghai, China). This cDNA served as the template for amplifying the *atg8-1* and *atg8-2* sequences through polymerase chain reaction (PCR). The resulting amplicons were ligated into the pMAT2075 expression vector at the *Xho* I and *Nhe* I restriction sites, generating the recombinant constructs pMAT2075-ATG8-1 and pMAT2075-ATG8-2. These plasmids were linearized via *Sma* I digestion and delivered into *M. circinelloides* host cells by electroporation, following the protocol established by Torres-Martínez et al. [26]. Primary transformants were screened on casamino acid minimal medium (MMC) solid medium (pH 3.2, 28 °C) supplemented with 200 μg/mL uracil where necessary. Genomic DNA from potential candidates was isolated using a DNA Fast Plant System Kit (Tiangen Biotech, Beijing, China) to confirm successful integration via PCR. Confirmed strains, designated as McATG8-1 and McATG8-2, were utilized for fermentation analysis, with a strain harboring the empty pMAT2075 vector (designated as Mc2075) serving as the negative control.

### 2.3. Media Compositions

Kendrick and Ratledge (K&R) seed medium was prepared by mixing Solution A and Solution B. Solution A contained 30.0 g/L glucose, 1.5 g/L MgSO_4_·7H_2_O, and 100 µL/L of a pre-formulated trace element solution (8.0 mg/L FeCl_3_·6H_2_O, 1.0 mg/L ZnSO_4_·7H_2_O, 0.1 mg/L CuSO_4_·5H_2_O, 0.1 mg/L MnSO_4_·5H_2_O, and 0.1 mg/L Co(NO_3_)_2_·6H_2_O). Solution B contained 3.3 g/L ammonium tartrate, 1.5 g/L yeast extract, 7.0 g/L KH_2_PO_4_, 0.1 g/L CaCl_2_·2H_2_O, and 2.0 g/L Na_2_HPO_4_. The modified K&R media for fermentation were formulated based on the seed medium matrix. The nitrogen-limited fermentation medium contained 80.0 g/L glucose, 2.0 g/L ammonium tartrate, and 1.5 g/L yeast extract, while the nitrogen-rich fermentation medium contained 80.0 g/L glucose, 20.0 g/L ammonium tartrate, and 15.0 g/L yeast extract, with all other components remaining identical to the seed medium.

### 2.4. Liquid Fermentation of McATG8 Overexpressing Strains

Verified strains Mc2075, McATG8-1, and McATG8-2 were each inoculated at a density of 1.0 × 10^7^ spores into 500 mL baffled flasks containing 100 mL of Kendrick and Ratledge (K&R) seed medium [27] (detailed in Section 2.3). A 10% (*v*/*v*) inoculum was transferred into 100 mL of modified K&R medium, supplemented with 80.0 g/L glucose, for a 96 h screening phase after a 24 h seed cultivation period at 28 °C with constant shaking at 130 rpm. The most promising transformants were subsequently scaled up in a 1.5 L fermenter (Dibier Bioengineering, Shanghai, China) in 1.0 L of the same modified medium. The temperature was maintained at 28 °C, with stirring and aeration rates fixed at 800 rpm and 1.5 vvm, respectively. This specific agitation and aeration regime was based on empirical optimization performed in our laboratory for *M. circinelloides* submerged cultivation, it maximizes oxygen transfer and nutrient mixing, which are essential for lipid biosynthesis, while maintaining the parameters below the threshold that induces severe mechanical disruption or loss of cell viability in filamentous mycelia. The culture pH was stabilized at 6.0 via automated titration using 2.0 M NaOH over the total fermentation duration of 120 h.

### 2.5. Biochemical Characterization of Cell Growth

Fresh mycelial samples were collected at designated sampling points by filtration through a Büchner funnel, and then freeze-dried. The mycelial biomass, expressed as the cell dry weight (CDW), was quantified gravimetrically. The residual glucose concentrations in the fermentation broth were determined using a commercial glucose oxidase peroxidase kit (Beijing Reagent Biotechnology Co., Ltd., Beijing, China). The indophenol blue method of Dang et al. [24] was used for colorimetric quantification of ammonium nitrogen in the fermentation broth at each sampling time point.

### 2.6. Determination of Lipid Content and Fatty Acid Composition

Lipid extraction and methylation were performed according to a previously described method [24]. Briefly, cells (15 mg) were subjected to acid hydrolysis by incubating them in 2 mL of 4 M hydrochloric acid (HCl) at 80 °C in a constant temperature water bath for 3 h, with vortexing performed every 30 min to ensure complete disruption. This acid treatment hydrolyzes structural polysaccharides (such as chitin and glucans), effectively disrupting the rigid fungal cell walls and releasing intracellular lipid droplets. Following hydrolysis and subsequent cooling to room temperature, a chloroform/methanol mixture (2:1, *v*/*v*) was added directly to the hydrolysate to partition and extract the lipids into the organic phase, with pentadecanoic acid (C15:0) added as an internal standard. The recovered lipids underwent methylation by incubation in a 10% (*w*/*w*) sulfuric acid/methanol solution at 60 °C for 3 h, followed by extraction with *n*-hexane.

The fatty acid methyl esters obtained from the samples were then subjected to gas chromatographic (GC) analysis to determine lipid composition and content, using a GC system fitted with a DB-Waxetr column (30 m × 0.32 mm, 0.25 μm; Agilent 123-3232, Santa Clara, CA, USA). The GC oven temperature was programmed as follows: initial hold at 80 °C for 10 min, then increase to 160 °C at a rate of 8 °C/min, subsequently increase to 220 °C at 4 °C/min, and finally hold at 220 °C for 2 min.

Total fatty acid (TFA) content was quantified using the internal standard method, and the resulting fatty acid mass was normalized against cell dry weight (CDW). Final TFA values were expressed as a percentage of CDW (% CDW). All measurements were performed in triplicate for each sample.

### 2.7. Gene Expression Analysis by RT-qPCR

Gene expression levels were analyzed using reverse transcription quantitative PCR (RT-qPCR). The recombinant strains McATG8-1T2 and McATG8-2T2, along with the control strain Mc2075, were cultivated under nitrogen-limited conditions. Mycelial samples were collected at selected fermentation time points, and total RNA was extracted using an appropriate RNA isolation kit (Sangon Biotech, Shanghai, China) according to the manufacturer’s guidelines. RNA quality and concentration were evaluated before downstream analysis. Purified RNA was subsequently reverse transcribed into complementary DNA (cDNA) using a commercial Evo M-MLV reverse transcription premix kit (Accurate Biotechnology Co., Ltd., Changsha, China).

RT-qPCR was performed using gene-specific primers targeting key genes involved in autophagy (*atg8-1*, *atg8-2*, and *atg1*), central carbon metabolism (hexokinase (*hk*)), lipid biosynthesis (ATP-citrate lyase (*acl*), acetyl-CoA carboxylase (*acc*), and fatty acid synthase (*fas*)), and the pentose phosphate pathway (PPP) (6-phosphogluconate dehydrogenase (*6pgdh*)). Amplification reactions were performed on a LightCycler 96 Real-Time PCR System (Roche Diagnostics GmbH, Mannheim, Germany). The thermal cycling program was set as follows: pre-denaturation at 95 °C for 30 s, followed by 40 cycles of denaturation at 95 °C for 5 s and annealing at 60 °C for 30 s. The specificity and amplification efficiency of all primer pairs were evaluated prior to the analysis, confirming their suitability for reliable relative quantification. All primer sequences utilized in this work are listed in the Supplementary Materials (Table S1). The 2^−ΔΔCt^ method [28] was used to calculate relative gene expression levels, with the housekeeping gene *actin* used as an internal reference for normalization. All experiments were conducted with three independent biological replicates to ensure reproducibility.

### 2.8. Ethanolamine Supplementation Experiments

Ethanolamine was used to evaluate the effect of PE precursor availability on cell growth and lipid accumulation. A 1.0 mL volume of a 5.0 M stock solution was added to 100 mL of fermentation medium at the onset of cultivation, yielding a final concentration of 50 mM. The recombinant and control strains were cultivated in a nitrogen-limited medium, and samples were collected at defined time intervals. Cell growth and lipid accumulation were determined by CDW measurement and TFA analysis, respectively, as described above.

### 2.9. Rapamycin Treatment Experiments

Rapamycin was first prepared as a 2 mM stock solution in dimethyl sulfoxide (DMSO). This stock solution was added into the fermentation medium at the beginning of cultivation to achieve a final concentration of 2 µM. Control groups received an equal volume of DMSO without rapamycin. All strains were cultivated in nitrogen-limited and nitrogen-rich media. The nitrogen concentration in the nitrogen-rich medium was five-fold higher than that in the nitrogen-limited medium. Samples were collected at defined time intervals to evaluate cell growth and lipid accumulation through CDW and TFA measurements, respectively.

### 2.10. Statistical Analysis

All experiments were performed in at least biological triplicate to ensure reproducibility, and data are presented as the mean ± standard deviation (SD). Statistical evaluation of differences between experimental groups was conducted using one-way analysis of variance (ANOVA). Homogeneity of variance was verified using Levene’s test prior to the comparison. When the overall ANOVA yielded significant differences (*p* ≤ 0.05), Fisher’s least significant difference (LSD) test was applied for pairwise post hoc multiple comparisons, and statistical groupings were designated according to Duncan’s multiple range test. If the overall ANOVA indicated no statistical significance, identical superscript letters were uniformly assigned across all groups. A value of *p* ≤ 0.05 was considered statistically significant for all evaluations.

## 3. Results

### 3.1. Construction of atg8 Overexpression Strains in M. circinelloides

**Figure 1:**
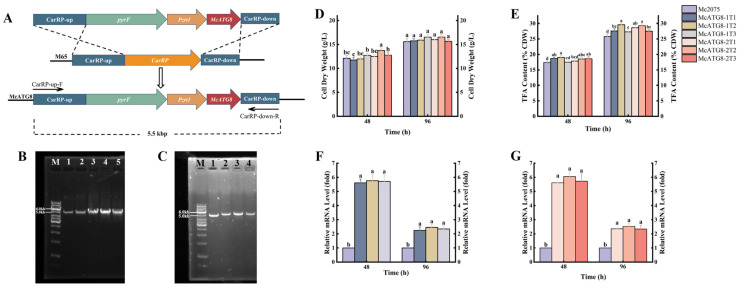
Overexpression of the *atg8* genes in *M. circinelloides* WJ11 and shake-flask cultivation performance of the overexpression strains McATG8-1 and McATG8-2 under nitrogen-limited conditions. (**A**) Homologous recombination strategy for the genomic integration of McATG8-1 and McATG8-2 under the control of the Pzrt1 promoter. Primer binding sites and anticipated PCR product sizes are shown. (**B**) PCR confirmation of the control strain Mc2075 (lanes 1–2) and positive transformants McATG8-1 (lanes 3–5). (**C**) PCR confirmation of the control strain Mc2075 (lane 1) and positive transformants McATG8-2 (lanes 2–4). Relevant fragment sizes are indicated in kilobases (kb). Lane M contained the GeneRuler DNA Ladder Mix (Thermo Fisher Scientific, Waltham, MA, USA). (**D**) CDW. (**E**) TFA content. (**F**) Relative transcription level of *atg8-1* in McATG8-1. (**G**) Relative transcription level of *atg8-2* in McATG8-2. Results are expressed as the mean ± SD of three biological replicates, with distinct superscript letters signifying statistical significance (*p* ≤ 0.05).

The cDNA lengths of the two *atg8* genes according to the annotated genome of *M. circinelloides* WJ11 are 354 bp (*atg8-1*) and 375 bp (*atg8-2*), respectively. Fragments of *atg8-1* and *atg8-2* were amplified from the *M. circinelloides* genome and cloned into the expression vector pMAT2075. Using a homologous recombination strategy, the *atg8* overexpression strains were constructed (Figure 1A). A strain harboring the empty vector pMAT2075 served as the negative control and was designated as Mc2075. Transformation was performed by electroporation, and recombinant strains were screened based on colony color, yielding transformants McATG8-1T1-T3 and McATG8-2T1-T3. PCR analysis of the transformants demonstrated that the control strain Mc2075 exhibited a band of approximately 5.1 kb, whereas bands of about 5.5 kb were observed in the McATG8 strains (Figure 1B,C). Given the relatively minor size differences in the PCR products, the mRNA expression levels of *atg8* in McATG8-1 and McATG8-2 were quantified and found to be consistently higher than in the control strain during shake-flask cultivation, thereby confirming the successful integration and active transcription of *atg8-1* and *atg8-2* within the transformant genomes (Figure 1F,G). The validated transformants were cultured in a nitrogen-limited medium in shake flasks. The CDW remained highly comparable between the transformants and the control strain Mc2075, except for a slight variation in McATG8-1T3 and McATG8-2T2 (Figure 1D), indicating that *atg8* overexpression neither impaired basal physiological functions nor significantly promoted growth. The TFA content was significantly elevated in the transformants, although the extent of the increase varied (Figure 1E). In transformant McATG8-1T2, it reached 19.0% at 48 h and 29.6% at 96 h (the end of fermentation), representing significant relative increases of 9.2% and 14.7%, respectively, compared with the control (17.4% at 48 h and 25.8% at 96 h). In transformant McATG8-2T2, it reached 18.5% at 48 h and 29.3% at 96 h, corresponding to significant relative increases of 6.3% and 13.5% compared with the control. Transformants McATG8-1T2 and McATG8-2T2 were therefore selected for further scaled-up cultivation in fermenters to investigate the effect of overexpressing the autophagy-related protein ATG8 on lipid accumulation in *M. circinelloides*.

### 3.2. Effect of atg8 Overexpression on Cell Growth in M. circinelloides During Nitrogen-Limited Cultivation

**Figure 2:**
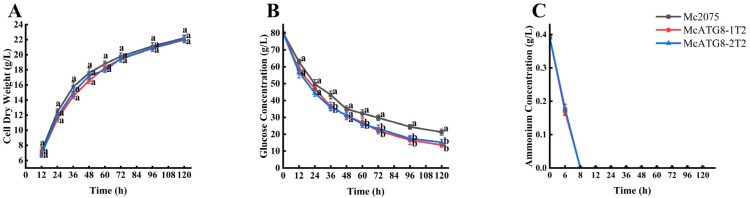
Cell growth and substrate consumption profiles of Mc2075, McATG8-1T2, and McATG8-2T2 during nitrogen-limited cultivation. (**A**) CDW. (**B**) Glucose concentration. (**C**) Ammonium ion concentration. Results are expressed as the mean ± SD of three biological replicates, with distinct superscript letters signifying statistical significance (*p* ≤ 0.05).

The best-performing transformants from the shake-flask screening, McATG8-1T2 and McATG8-2T2, were cultured in scale-up in fermenters alongside the Mc2075 control to investigate how *atg8* overexpression modulates the growth and metabolism of *M. circinelloides*. Fermentation kinetics were subsequently characterized by tracking CDW accumulation and nutrient consumption. These results showed that the recombinant strains McATG8-1T2 and McATG8-2T2 exhibited growth trajectories comparable to that of the Mc2075 strain during the entire cultivation phase (Figure 2A). These findings confirmed that upregulating the autophagy-related gene *atg8* did not have a detrimental impact on basal cell growth or CDW yield in *M. circinelloides*, aligning with the shake-flask data.

All strains exhibited highly consistent rates of nitrogen source consumption. The ammonium ion concentration in the medium decreased rapidly and was completely depleted within approximately 8 h of fermentation (Figure 2C). This result indicates that all strains entered nitrogen starvation stress simultaneously. Given that the nitrogen source was exhausted at an early stage (8 h) while the fermentation lasted for 120 h, the cultures experienced nitrogen restriction for over 90% of the total cultivation timeframe. This prolonged nitrogen starvation environment likely drove a shift in cellular metabolic patterns, thereby providing the necessary inducing conditions for rapid lipid accumulation and ATG8-mediated resource reallocation. Monitoring of carbon source metabolism further showed that all strains exhibited vigorous glucose uptake during the rapid growth phase in the first 24 h of fermentation (Figure 2B). The glucose consumption rates of the two recombinant strains were significantly faster than that of the control strain starting from 36 h (coinciding with the onset of the lipid accumulation phase). At the end of fermentation, the residual glucose concentration in the medium remained at 21.3 g/L in the control strain, whereas it decreased to 13.6 g/L in McATG8-1T2 and 15.2 g/L in McATG8-2T2. These results suggest that carbon was sufficiently available throughout fermentation and that *atg8* overexpression promoted substrate utilization during the middle and late stages of cultivation.

### 3.3. Effect of atg8 Overexpression on Lipid Accumulation in M. circinelloides During Nitrogen-Limited Cultivation

**Figure 3:**
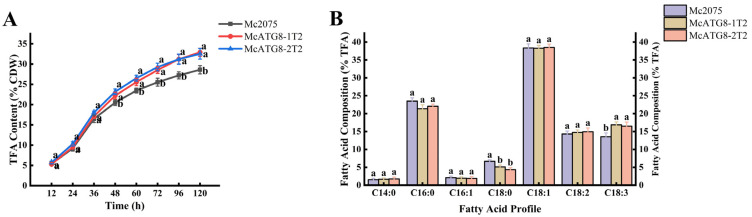
Lipid accumulation of Mc2075, McATG8-1T2, and McATG8-2T2 during nitrogen-limited cultivation. (**A**) TFA content. (**B**) Fatty acid composition at 120 h. Results are expressed as the mean ± SD of three biological replicates, with distinct superscript letters signifying statistical significance (*p* ≤ 0.05).

The TFA content of all strains continued to increase with fermentation time during the nitrogen starvation phase (12–120 h) (Figure 3A). The differences in TFA content between the recombinant strains and the control strain remained small at the early fermentation stages (12 and 24 h) and at the onset of rapid lipid accumulation (36 h), indicating that the initial metabolic state of each strain was similar during the initial stage of lipid synthesis. Lipid accumulation in the recombinant strains McATG8-1T2 and McATG8-2T2 subsequently significantly outpaced that of the control. At the end of fermentation (120 h), the TFA content of the control strain Mc2075 reached 28.6%, while those of McATG8-1T2 and McATG8-2T2 reached 32.9% and 32.5%, respectively, representing relative increases of 15.0% and 13.7% compared with the control. These results suggest that the higher glucose consumption rate of the recombinant strains coincided with their enhanced lipid accumulation, supported by the observation that the recombinant strains consumed glucose significantly faster from 36 h onward (Figure 2B). The additional glucose consumed was likely directed toward lipid synthesis based on the TFA data, suggesting that *atg8* overexpression may promote carbon flux toward lipid accumulation.

Differences in fatty acid composition were observed between McATG8-1T2, McATG8-2T2, and the control strain Mc2075 at the end of fermentation. The proportions of saturated fatty acids, mainly palmitic acid, showed decreasing trends in the recombinant strains, with relative reductions of 9.1% and 6.2%, respectively, compared with Mc2075 (23.5%). In contrast, the proportion of the polyunsaturated fatty acid GLA significantly increased, reaching approximately 16.9% and 16.5% in McATG8-1T2 and McATG8-2T2, respectively, at the end of fermentation (Figure 3B), representing relative increases of 25.2% and 22.0% compared with Mc2075 (13.5%). The proportions of oleic and linoleic acids showed no significant changes and remained relatively stable.

### 3.4. Expression of Key Genes Involved in Autophagy and Fatty Acid Biosynthesis Mediated by atg8 Overexpression During Nitrogen-Limited Cultivation

**Figure 4:**
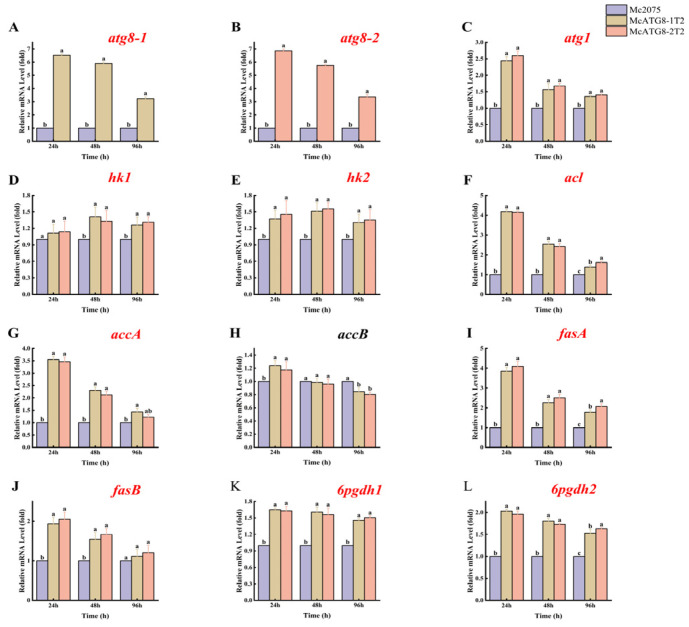
Transcriptional dynamics of key target genes in Mc2075 and *atg8*-overexpressing transformants at 24, 48, and 96 h during nitrogen-limited cultivation. (**A**) *atg8-1* and (**B**) *atg8-2*, autophagy-related gene 8; (**C**) *atg1*, autophagy-related gene 1; (**D**) *hk1* and (**E**) *hk2*, hexokinase; (**F**) *acl*, ATP-citrate lyase; (**G**) *accA* and (**H**) *accB*, acetyl-CoA carboxylase; (**I**) *fasA* and (**J**) *fasB*, fatty acid synthase; (**K**) *6pgdh1* and (**L**) *6pgdh2*, 6-phosphogluconate dehydrogenase. Red represents significant up-regulation. Results are expressed as the mean ± SD of three biological replicates, with distinct superscript letters signifying statistical significance (*p* ≤ 0.05).

Lipid analysis revealed that the TFA content in the *atg8*-overexpressing transformants differed markedly from that of the control. Key genes involved in autophagy, central carbon metabolism, and lipid synthesis were identified from the *M. circinelloides* WJ11 genome, and their transcriptional responses to *atg8* overexpression were analyzed to explore the mechanisms of intracellular resource reallocation (Figure 4). The persistently elevated transcripts of *atg8-1* and *atg8-2* throughout the entire cultivation cycle provided definitive proof for the successful incorporation of the *atg8* cassette into the *M. circinelloides* genome. The transcription level of *atg1* (Figure 4C), a key kinase in the autophagy initiation complex, was also significantly upregulated in the recombinant strains, indicating that *atg8* overexpression activated the autophagy pathway. Key genes driving central carbon metabolism and lipid biosynthesis exhibited pronounced transcriptional induction. The expression of *hk*, the rate-limiting enzyme of glycolysis, was markedly elevated (Figure 4D,E), which is consistent with the rapid glucose consumption phenotype of the recombinant strains. The transcription of the *acl* gene was also significantly upregulated (Figure 4F) in the recombinant strains. Consistently, the transcription levels of *acc* (Figure 4G) and *fas* (Figure 4I,J) were both significantly increased, and their expression trends showed a strong positive correlation with the increase in intracellular TFA content. The supply of reducing power was also investigated to explain the observed increase in the GLA proportion within the fatty acid profile. The conversion of fatty acids to GLA via desaturation is strongly dependent on NADPH. Therefore the mRNA expression levels of the *6pgdh* genes, which encode a key rate-limiting enzyme of the PPP, were measured and found to be significantly upregulated in the recombinant strains (Figure 4K,L). This suggests that the engineered strains enhanced the PPP to provide sufficient NADPH to support the energy-intensive fatty acid desaturation reactions, which is also consistent with the rapid glucose consumption phenotype of the recombinant strains (Figure 2B).

### 3.5. Effects of Exogenous Ethanolamine Supplementation on Cell Growth and Lipid Accumulation in atg8-Overexpressing Strains of M. circinelloides During Nitrogen-Limited Cultivation

**Figure 5:**
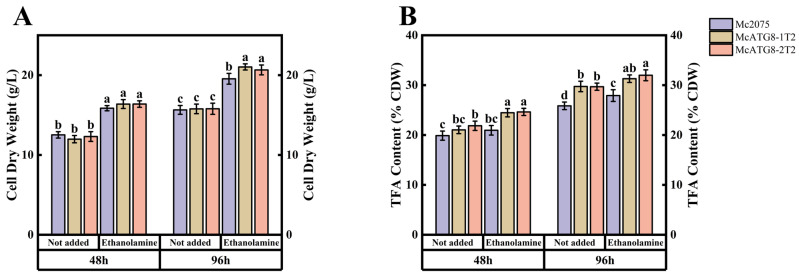
Effect of ethanolamine supplementation on cell growth and lipid accumulation in *M. circinelloides* during nitrogen-limited cultivation. (**A**) CDW. (**B**) TFA content. Results are expressed as the mean ± SD of three biological replicates, with distinct superscript letters signifying statistical significance (*p* ≤ 0.05).

To investigate whether intracellular phosphatidylethanolamine (PE) availability influences ATG8-dependent lipid synthesis, exogenous ethanolamine, a precursor for PE biosynthesis, was added to the fermentation medium. The results demonstrated that ethanolamine treatment significantly increased the CDW of all strains compared with their CDWs under conventional nitrogen-limited fermentation conditions (Figure 5A). At the completion of fermentation at 96 h, the CDW of Mc2075 significantly increased by 3.8% (from 15.7 to 19.5 g/L), that of McATG8-1T2 increased by 5.2% (from 15.8 to 21.0 g/L), whereas that of McATG8-2T2 increased by 4.9% (from 15.8 to 20.7 g/L). The CDWs of the ethanolamine-supplemented McATG8-1T2 and McATG8-2T2 groups significantly increased by 7.7% and 6.2% at 96 h, respectively, compared with the control strain Mc2075. Ethanolamine treatment also significantly enhanced the lipid synthesis capacity of all strains at 96 h (Figure 5B). Specifically, ethanolamine treatment increased the TFA content in Mc2075 by 2.0% (from 25.9% to 27.9%) compared with its content under conventional nitrogen-limited fermentation conditions, whereas the corresponding increases in McATG8-1T2 and McATG8-2T2 were 1.6% (from 29.7% to 31.3%) and 2.3% (from 29.7% to 32.0%), respectively. In the ethanolamine-supplemented McATG8-1T2 and McATG8-2T2 groups, the TFA content significantly increased by 12.2% and 14.6%, respectively, in comparison with the control strain Mc2075 at 96 h. The combined effect of *atg8* overexpression and exogenous ethanolamine supplementation significantly enhanced the TFA content in *M. circinelloides* under nitrogen limitation conditions, with TFA levels in McATG8-1T2 and McATG8-2T2 reaching 20.8% and 23.6% above that of Mc2075, respectively.

### 3.6. Effects of Exogenous Addition of Rapamycin on Cell Growth and Lipid Accumulation in atg8-Overexpressing Strains of M. circinelloides

**Figure 6:**
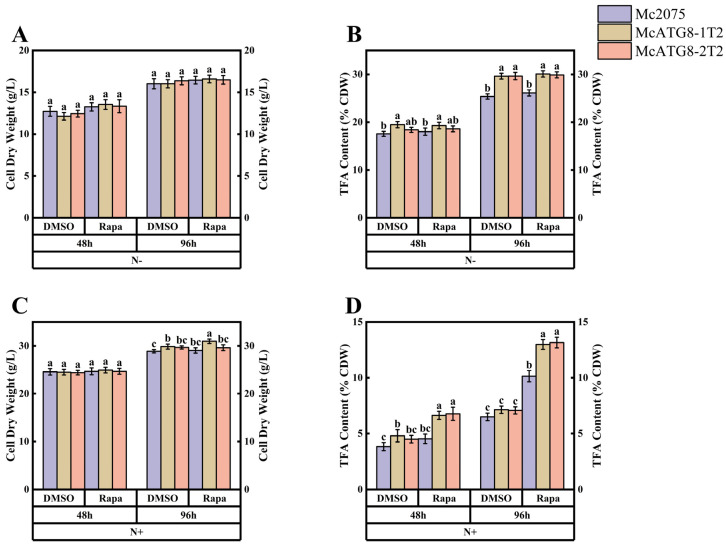
Cell growth and lipid accumulation of Mc2075, McATG8-1T2, and McATG8-2T2 in rapamycin-supplemented medium under nitrogen-limited and nitrogen-rich conditions. (**A**) CDW under nitrogen-limited conditions. (**B**) TFA content under nitrogen-limited conditions. (**C**) CDW under nitrogen-rich conditions. (**D**) TFA content under nitrogen-rich conditions. Results are expressed as the mean ± SD of three biological replicates, with distinct superscript letters signifying statistical significance (*p* ≤ 0.05).

Dang et al. reported that rapamycin specifically inhibits TORC1 activity in *M. circinelloides*, thereby relieving TORC1 inhibition of autophagy and promoting fatty acid biosynthesis [24]. ATG8, a key factor in autophagosome membrane formation, functions downstream of the TORC1 signaling pathway. Rapamycin was exogenously added to the fermentation medium of the recombinant strains McATG8-1T2 and McATG8-2T2 under nitrogen-limited and nitrogen-rich conditions to investigate whether ATG8-mediated lipid accumulation in *M. circinelloides* is regulated by upstream TORC1 signaling and to assess whether further enhancing autophagy can continue to improve resource reallocation and lipid accumulation. The CDW and TFA contents at 48 and 96 h remained largely unchanged across all the rapamycin-treated groups compared with the control group without rapamycin (DMSO group) under nitrogen-limited conditions (Figure 6A,B). The TFA contents of the recombinant strains McATG8-1T2 (30.1%) and McATG8-2T2 (29.9%) were 4.0%and 3.8% higher than that of the control strain Mc2075 (26.1%), respectively, under rapamycin treatment at 96 h. These differences are highly consistent with the lipid accumulation characteristics observed under untreated conditions in the previous section (Figure 3A). Overall, rapamycin did not exert an additional promoting effect on cell growth and lipid accumulation in synergy with *atg8* overexpression under nitrogen-limited conditions.

In contrast, the addition of rapamycin significantly altered the lipid accumulation pattern under nitrogen-rich conditions at 48 and 96 h (Figure 6D). TFA contents were significantly elevated following rapamycin treatment, whereas CDWs remained statistically unchanged across all strains. Rapamycin treatment increased the TFA content of Mc2075 by 3.6% (from 6.5% to 10.1%), that of McATG8-1T2 by 5.9% (from 7.1% to 13.0%), and that of McATG8-2T2 by 6.1% (from 7.1% to 13.2%) compared with the DMSO control group at 96 h. Notably, under rapamycin treatment, the TFA contents of the recombinant strains were significantly higher than those of the control strain, with relative increases of 28.7% and 30.7%, respectively. The TFA contents of McATG8-1T2 and McATG8-2T2 showed significant increases of 99.9% and 103.1%, respectively, compared with the control strain without rapamycin treatment.

## 4. Discussion

**Figure 7:**
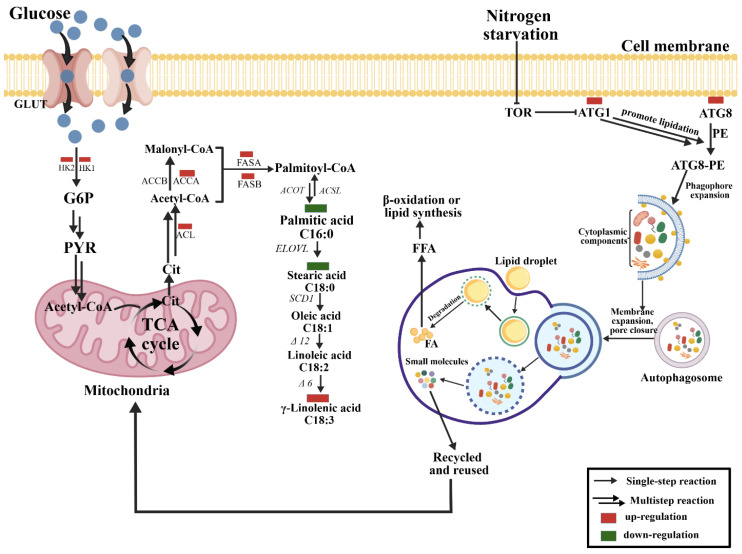
Synergistic regulation of lipid accumulation and autophagy by *atg8* overexpression in *M. circinelloides* under nitrogen starvation. ACC, acetyl-CoA carboxylase; GLUT, glucose transporter; HK, hexokinase; G6P, glucose-6-phosphate; PYR, pyruvate; TCA cycle, tricarboxylic acid cycle; ACOT, acyl-CoA thioesterase; FAS, fatty acid synthase; ACSL, long-chain acyl-CoA synthetase; ELOVL, elongase of very long chain fatty acids; SCD1, stearoyl-CoA desaturase 1; FADS12, fatty acid *Δ*12 desaturase; FADS6, fatty acid *Δ*6 desaturase. Red and green indicate up-regulation and down-regulation. This figure was created with BioGDP.com [32].

The homologous overexpression of autophagy-related genes *atg8-1* and *atg8-2* in *M. circinelloides* significantly increased the TFA content during nitrogen-limited cultivation without adversely affecting cell growth. RT-qPCR and metabolite analyses further confirmed that the high-lipid phenotype of the recombinant strains was closely associated with autophagy-mediated intracellular carbon skeleton reallocation and metabolic flux remodeling. Traditionally, autophagy is regarded as a conserved catabolic process [29], and excessive autophagy could lead to cell death, thereby resulting in a decrease in CDW [30,31]. However, the CDW of the recombinant strains did not differ significantly from that of the control strain in this study. This observation indicates that the enhanced autophagy mediated by ATG8 does not simply represent a depletion of cellular components, but rather an efficient metabolic switching strategy. By degrading non-essential components such as ribosomes and proteins, the cell redirects their carbon skeletons toward the synthesis of energy-dense TAGs [12]. The net increase in lipid mass successfully compensates for the mass loss caused by intracellular macromolecule degradation (Figure 7). The RT-qPCR results further revealed that alongside the sustained high expression of the target gene *atg8* throughout the fermentation period, *atg1* was also significantly upregulated in the recombinant strains. This suggests that overexpression of *atg8* triggers a systemic activation of the entire autophagic pathway, from ATG1-mediated initiation to ATG8-PE conjugation during autophagosome elongation. Collectively, these results indicate that under nitrogen starvation stress, *atg8* and its encoded protein ATG8 may play an important role in autophagy-mediated resource reallocation.

The high glucose consumption observed in the recombinant strains indicated that overexpression of *atg8* exerted a significant regulatory effect on central carbon metabolism. This process may be driven by a dual mechanism involving autophagy-mediated endogenous recycling and exogenous uptake. Endogenous recycling via autophagy directly recovers carbon skeletons by degrading non-essential macromolecules, thereby supplying acetyl-CoA, a key precursor for lipid synthesis [12]. In parallel, active exogenous uptake ensures the continuous and efficient internalization of glucose, which is primarily metabolized via glycolysis and the PPP. The RT-qPCR results further support this metabolic flux remodeling mechanism: *hk* was significantly upregulated, which explains the active uptake of exogenous glucose by the cells, whereas the upregulation of *acl*, acting as a crucial bridge between carbon metabolism and lipid synthesis, ensures that carbon flux entering the mitochondria, whether derived from glycolysis or autophagy-mediated recycling, is efficiently converted into cytosolic acetyl-CoA.

The ample supply of precursors supported the global activation of lipid synthesis. RT-qPCR revealed that the gene encoding the rate-limiting enzyme of fatty acid synthesis, *accA*, as well as the key gene *fas*, were both significantly upregulated, showing a strong positive correlation with the increase in TFA content. Notably, a marked shift in fatty acid composition was observed, from saturated palmitic acid (C16:0) to polyunsaturated GLA (C18:3). Desaturation and chain elongation of lipids are highly energy-demanding processes [33,34]. RT-qPCR showed that the transcript level of *6pgdh*, a key enzyme gene of the PPP, was significantly upregulated, indicating activated NADPH generation pathways. The ample supply of reducing power not only supports the catalytic activity of fatty acid synthase but also relieves the cofactor limitation of energy-intensive desaturation reactions, thereby driving the conversion of fatty acids toward long-chain polyunsaturated forms. Furthermore, autophagy may also actively mediate the turnover of membrane phospholipids, thereby recycling membrane lipids rich in unsaturated fatty acids back into TAGs [35]. This process not only further optimizes the cellular fatty acid profile but also extends the metabolic implications of the aforementioned endogenous recycling mechanism. Meanwhile, due to their multiple double bonds, PUFAs are highly susceptible to attack by intracellular free radicals. Enhanced autophagic activity can effectively clear damaged organelles and reduce intracellular reactive oxygen species levels, thereby mitigating lipid peroxidation-induced damage to polyunsaturated fatty acids and ensuring that their high levels are maintained, even during the late stage of fermentation.

Validation experiments were performed using exogenous supplementation with rapamycin and ethanolamine to investigate the role of ATG8 within the autophagic regulatory network and its functional constraints. Regarding upstream regulation, rapamycin inhibits the TORC1 complex under nitrogen-rich conditions [36], thereby mimicking starvation signals and inducing autophagy. In this study, rapamycin treatment led to an increase in lipid content in the control strain, while the *atg8*-overexpressing strains exhibited a further enhancement in lipid accumulation. This finding demonstrates that when the upstream TORC1 signaling pathway is inhibited and autophagy repression is relieved, the abundance of the downstream effector protein ATG8 becomes a major limiting factor for autophagic flux. In contrast, rapamycin treatment did not produce an additional promoting effect under nitrogen-limited conditions, suggesting that nitrogen depletion alone was sufficient to strongly inhibit TORC1 activity and maintain autophagy in a highly active state. This result further supports the conclusion that nitrogen-limitation-induced lipid accumulation is closely associated with TORC1 inhibition and autophagic activation. Further analysis revealed that although rapamycin activated autophagy under nitrogen-replete conditions, the total lipid accumulation remained significantly lower than that in the nitrogen-limited cultivation system. This suggests that the regulation of lipid synthesis by nitrogen availability extends beyond the TORC1 pathway and involves global metabolic reallocation. An ample nitrogen supply can maintain active protein synthesis and cell growth, thereby competitively diverting central carbon metabolites. In contrast, under nitrogen starvation, not only was the TORC1 pathway suppressed but also global protein translation [37], resulting in substantial conservation of carbon skeletons and energy that would otherwise be used for growth, and redirecting them toward the fatty acid synthesis pathway.

The ethanolamine supplementation experiments further revealed the biochemical constraints on ATG8 function. PE is an essential substrate for ATG8 lipidation and serves as an indispensable component of the ATG8-PE conjugation system during autophagosome formation [38,39]. Previous studies have shown that exogenous ethanolamine supplementation can expand the intracellular PE pool and positively regulate autophagic activity [40]. Intracellular PE abundance was therefore hypothesized to be a limiting factor for ATG8 function and consequently for TFA accumulation during nitrogen-limited cultivation. Excessive accumulation of ATG8 protein in the recombinant strains may lead to a relative scarcity of the endogenous PE pool, thereby creating a substrate supply bottleneck. The experimental results showed that exogenous supplementation with ethanolamine, which expands the intracellular PE pool, significantly increased the lipid yield in the recombinant strains. This indicated that the availability of endogenous PE became a secondary rate-limiting factor restricting autophagic flux and lipid accumulation efficiency under the genetic background of *atg8* overexpression. Therefore, maintaining the stoichiometric balance between ATG8 and its lipid substrate PE is crucial for maximizing autophagy-mediated metabolic flux. An adequate supply of PE may also provide essential structural components and lipid precursors for lipid droplet biogenesis and expansion by promoting the dynamic turnover of membrane systems while enhancing autophagic flux [41]. Studies have shown that PE not only serves as a key constituent of the phospholipid monolayer on the surface of lipid droplets [42,43] but also that increased PE levels can promote lipid droplet fusion and size enlargement [44]. There is also a metabolic flux switch between membrane phospholipids and storage lipids [45,46]. Therefore, in the context of *atg8* overexpression combined with PE supplementation, an adequate PE supply may indirectly expand the cellular storage capacity for neutral lipids through the aforementioned mechanisms. In summary, exogenous ethanolamine supplementation not only effectively alleviated the potential substrate limitation but also acted synergistically with *atg8* overexpression to further promote lipid accumulation. This not only confirms that autophagy positively regulates lipid accumulation in *M. circinelloides* but, more importantly, it also reveals that intracellular PE can serve as a key molecular hub connecting autophagic flux and lipid metabolism.

Both homologous *atg8* genes identified in this study, *atg8-1* and *atg8-2*, produced similar lipid-promoting phenotypes upon overexpression. The overexpression of both genes resulted in comparable increases in total lipid content and similar changes in fatty acid composition. This indicates that during the evolution of *M. circinelloides*, these two homologous genes, generated by gene duplication, have retained a high degree of functional redundancy. This redundancy may confer greater robustness to the strain under complex environmental stress [47], ensuring that even if one gene copy is impaired, the cell can still maintain the necessary autophagic activity to cope with nitrogen starvation using the other copy. This also provides multiple alternative and equivalent genetic manipulation targets for subsequent metabolic engineering efforts.

## 5. Conclusions

Overexpression of the autophagy-related genes *atg8-1* and *atg8-2* significantly improved total lipid and γ-linolenic acid yields in *M. circinelloides* under nitrogen-limited conditions without compromising cell growth. By facilitating efficient intracellular resource reallocation and coordinating with an expanded phosphatidylethanolamine pool, the enhanced autophagic flux promoted carbon redirection toward lipid biosynthesis. Overall, our results demonstrate that ATG8-mediated autophagy enhances lipid accumulation and acts as a key factor determining lipid synthesis in the oleaginous fungus *M. circinelloides*. These findings provide promising directions for the biotechnological application of *M. circinelloides* in the production of microbial lipids and γ-linolenic acid.

## Data Availability

The original contributions presented in this study are included in the article/Supplementary Material. Further inquiries can be directed to the corresponding authors.

## Supplementary Materials

The following supporting information can be downloaded at: https://www.mdpi.com/article/10.3390/jof12060410/s1. Table S1: List of primers used in this study.
