# Supplementary material for: The Role of ATG8 in Promoting Lipid Accumulation in the Oleaginous Fungus Mucor circinelloides During Nitrogen Limitation
Source: J Fungi (Basel). 2026 Jun 4;12(6):410. doi: 10.3390/jof12060410 (PMC13300868; doi:10.3390/jof12060410)
Supplement: Supplementary file 1 [file jof-12-00410-s001.zip › jof-4299972-supplementary.pdf]

Supplementary Materials

The Role of ATG8 in Promoting Lipid Accumulation in the Oleaginous Fungus *Mucor circinelloides* during Nitrogen Limitation

Hequn Li [a], Hongjuan Yuan[a], Bushra Iqbal[a], Tianyu Wang[a], Zhen Wang [b,*] and Huaiyuan Zhang[a,*].

a. Colin Ratledge Center for Microbial Lipids, School of Agriculture, Engineering and Food Science, Shandong University of Technology, 266 Xincun West Road, Zibo, Shandong, 255000, People's Republic of China.

b. School of Public Health, Qilu Medical University, Zibo, Shandong, 255300, People's Republic of China

***Corresponding Authors**

Huaiyuan Zhang

Email: zhyuan004@126.com

Zhen Wang

Email: wangzhen72@qlmu.edu.cn

Authors

Hequn Li

Email: 19811715276@163.com

Hongjuan Yuan

Email: y17663072167@163.com

Bushra Iqbal

Email: bushra.iqbal1212@gmail.com

Tianyu Wang

Email: 19819139268@163.com

Table S1 List of primers used in this study

Primers Name	Sequences (5'-3')	Annotation
NheI-F- ATG8-1	CTAGCTAGCATGCGTTCCAAGTTTAAAG	*atg8-1* fragment cloning
XhoI-R- ATG8-1	CCGCTCGAGTTAAAAGCCAAATGTATTTTC	
NheI-F- ATG8-2	CTAGCTAGCATGACTACCACATCAAAGCCTGC	*atg8-2* fragment cloning
XhoI-R- ATG8-2	CCGCTCGAGTCATAAGGCACAACCAAAGGTATT	
CarRP-up-F	GATAAGCATAAACCAGATCTGC	Checking for *atg8* overexpression
CarRP-down-R	GTATCTGACATAGTCGAGCTTC	
Actin-F	GATGAAGCCCAATCCAAGAGAGGT	RT-PCR for *actin*
Actin-R	TCTTCTCACGGTTGGACTTGGG	
ATG8-1-RT-F	ACGTAAAGCTGAAGCAGAGCGA	RT-PCR for *atg8-1*
ATG8-1-RT-R	TCCACCTTTTCACAAATCACAGGA	
ATG8-2-RT-F	TCCAAGCGAATCCTCAGCAAG	RT-PCR for *atg8-2*
ATG8-2-RT-R	AGTACTTTTGCTTGGCCATCCT	
ATG1-RT-F	CGAGTGTTGAAGCAAAGCCTGA	RT-PCR for *atg1*
ATG1-RT-R	GGCACTCTCATTTGTAGCATGG	
HK1-RT-F	TGCGATCCGTAGTGCTGCTT	RT-PCR for *hk1*
HK1-RT-R	TGCCAAACCAGTTGCGAAGG	
HK2-RT-F	ACCATGATCCCTTCCTATGT	RT-PCR for *hk2*
HK2-RT-R	CTTTGTTGTCTCGGTGGTAG	
ACL-RT-F	GGAAAAGAAATCAAGATTGAAAGAACGACTG	RT-PCR for *acl*
ACL-RT-R	AGCCTTGGCATCAACATCACC	
FAS A-RT-F	ACTGGTATCCGTTTCATTGAGCCTG	RT-PCR for *fas* A
FAS A-RT-R	AAATGTCTGCCTTTTCTCCCTGTTGA	
FAS B-RT-F	GGTCTTCGTTTCATTGACCCCACTG	RT-PCR for *fas* B
FAS B-RT-R	GACATCAGCCTTGTCACCTTGTTGG	
ACC A-RT-F	CTTTTGGGCAATGTCACGCA	RT-PCR for *acc* A
ACC A-RT-R	GGACAGCAACCATAGCACCT	
ACC B-RT-F	TCAGTCTACGGTGGTGGATCT	RT-PCR for *acc* B
ACC B-RT-R	CGTGGTAGAAAAAGTGGGGC	
6PGDH1-RT-F	GATGGTTCACAACGGTATTGAATACGGA	RT-PCR for *6pgdh1*
6PGDH1-RT-R	CGATCAAGAAAGAATCCAATTCACCCTTG	
6PGDH2-RT-F	GCACAACGGTATTGAGTACGGC	RT-PCR for *6pgdh1*
6PGDH2-RT-R	CCCTTATTCCATTCATCAAAGACATC	
